# How nature nurtures: Amygdala activity decreases as the result of a one-hour walk in nature

**DOI:** 10.1038/s41380-022-01720-6

**Published:** 2022-09-05

**Authors:** Sonja Sudimac, Vera Sale, Simone Kühn

**Affiliations:** 1 Max Planck Institute for Human Development, Lise Meitner Group for Environmental Neuroscience, Lentzeallee 94, 14195 Berlin, Germany; 2 Max Planck Institute for Human Development, Max Planck Dahlem Campus of Cognition (MPDCC), Lentzeallee 94, 14195 Berlin, Germany; 3 Max Planck Institute for Human Development, International Max Planck Research School on the Life Course (LIFE), Lentzeallee 94, 14195 Berlin, Germany; 4 University Medical Center Hamburg-Eppendorf, Department of Psychiatry and Psychotherapy, Martinistr. 52, 20251 Hamburg, Germany; 5 Max Planck UCL Centre for Computational Psychiatry and Ageing Research Berlin, Germany and London, UK, Lentzeallee 94, 14195 Berlin, Germany

**Keywords:** Neuroscience, Schizophrenia, Psychology

## Abstract

Since living in cities is associated with an increased risk for mental disorders such as anxiety disorders, depression, and schizophrenia, it is essential to understand how exposure to urban and natural environments affects mental health and the brain. It has been shown that the amygdala is more activated during a stress task in urban compared to rural dwellers. However, no study so far has examined the causal effects of natural and urban environments on stress-related brain mechanisms. To address this question, we conducted an intervention study to investigate changes in stress-related brain regions as an effect of a one-hour walk in an urban (busy street) vs. natural environment (forest). Brain activation was measured in 63 healthy participants, before and after the walk, using a fearful faces task and a social stress task. Our findings reveal that amygdala activation decreases after the walk in nature, whereas it remains stable after the walk in an urban environment. These results suggest that going for a walk in nature can have salutogenic effects on stress-related brain regions, and consequently, it may act as a preventive measure against mental strain and potentially disease. Given rapidly increasing urbanization, the present results may influence urban planning to create more accessible green areas and to adapt urban environments in a way that will be beneficial for citizens’ mental health.

## Introduction

The human brain is shaped by its surroundings. Increasing urbanization has been one of the recent major changes in our environment, resulting in more than half of the world’s population currently living in cities, projected to increase to 68% by 2050 [1].

Even though urbanization has many advantages, living in a city is a well-known risk factor for mental health [2]. Mental health problems like anxiety, mood disorders, major depression, and schizophrenia are up to 56% more common in urban compared to rural environments [3]. It has been suggested that urban upbringing is the most important environmental factor for developing schizophrenia [4], accounting for more than 30% of schizophrenia incidence [5]. Since there is a consistent dose-response relationship between schizophrenia and urban environment, even when controlling for possible confounders such as sociodemographic factors, family history, drug abuse, and size of social network [4], the hypothesis is that urban environment is related to higher schizophrenia incidence through increased social stress [6, 7].

On the other hand, exposure to nature provides attentional restoration and stress relief [8, 9]. The biophilia hypothesis states that humans feel an innate tendency to connect with nature since this attitude is rooted in our evolutionary history [10, 11]. Research about the beneficial effects of nature has been mainly motivated by two theoretical frameworks − Attention Restoration Theory (ART) [12] and Stress Recovery Theory (SRT) [13], that explain the psychological benefits of nature from different perspectives. ART focuses on cognitive restoration through nature exposure. The notion is that nature invokes involuntary attention allowing voluntary attention processes to recover [14]. SRT, on the other hand, emphasizes affective responses in contact with nature, that lead to restoration. According to SRT, the restorative process is related to the stress-reducing capacity of natural environments that involves an increase in positive emotions as well as a decrease in arousal and negative emotions such as fear [9, 13].

A growing body of empirical research has demonstrated the cognitive and affective benefits of exposure to natural environments. Spending time in nature can improve working memory capacity [15], restore directed attention [8] as well as reduce negative emotions and stress [16–18]. The evidence of nature’s beneficial effects on stress has been observed not only in psychological assessments, but also in physiological indicators of stress, namely in decreases in heart rate, blood pressure, and stress-related hormone cortisol [19, 20].

Even though the beneficial effects of nature exposure have been repeatedly shown, the neural underpinnings of these effects are unknown. In a seminal cross-sectional study, the amygdala has been shown to be more activated during a social stress task in urban compared to rural dwellers [21]. Nevertheless, intervention studies are needed to demonstrate the causal effects of natural and urban environments on the brain. In a single functional magnetic resonance imaging (fMRI) intervention study conducted so far it was shown that a 90-minute walk in nature decreased self-reported rumination and activity in the subgenual prefrontal cortex (sgPFC), associated with rumination, whereas there was no change after the urban walk [22].

However, to the best of our knowledge, there has been no fMRI intervention study examining the causal effects of exposure to urban vs. natural environments on stress-related brain regions. And importantly, the previous findings do not disentangle whether stress-relief after being in nature is the result of exposure to the natural environment itself or merely of the absence of detrimental urban effects. To address these questions, we conducted an fMRI intervention study investigating brain activity before and after a one-hour exposure to natural versus urban environments. We hypothesized that stress-related brain regions would be less activated after exposure to the natural compared to the urban environment, relative to the baseline activation before the walk. A-priori defined and preregistered (https://aspredicted.org/tm629.pdf) brain regions of interest (ROI) included amygdala, anterior cingulate cortex (ACC), and dorsolateral prefrontal cortex (dlPFC).

## Materials and methods

### Participants

Participants were recruited from the Castellum database of the Max Planck Institute for Human Development in Berlin, via mailing lists of universities in Berlin, and through the online platform *ebay-kleineanzeigen.de*. Participants were told that they would take part in an MRI study and that they would go for a walk, but were not informed about the research question of the study. All participants were fluent in the German language, right-handed, and were not diagnosed with any psychological or neurological disorders. A sample size estimation using G*Power resulted in the need of 54 participants to enable a medium effect size. We tested 9 participants more to ensure that potential drop outs would not reduce the sample size below the number we decided on. The final sample consisted of 63 participants (29 females, total mean age = 27.21 years, *SD* = 6.61, age-range = 18−47 years). The participants were pseudo-randomly assigned either to a nature (32 participants) or an urban walk (31 participants), while controlling that men and women were equally distributed in both environments. During randomization, it was also controlled that the number of afternoon walks were equally distributed between conditions. An overview over the control variables in the two conditions is shown in Supplementary Table 1.

The study was approved by the Local Psychological Ethical Committee at the Center for Psychosocial Medicine at University Medical Center Hamburg-Eppendorf in Hamburg, Germany (LPEK-0054). We obtained written informed consent from all participants and they received monetary compensation for the participation in the study.

### Study procedure

**Fig. 1:**
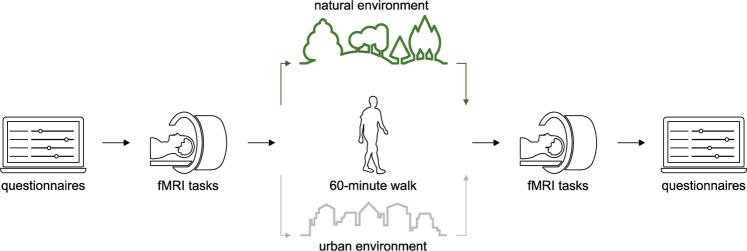
Flowchart of the study procedure. Before the walk participants filled out questionnaires and underwent the fMRI scanning procedure, which included the Fearful Faces Task and the Montreal Imaging Stress Task. Subsequently, each participant was randomly assigned to a 60-min walk, in either a natural or urban environment. After the walk, the participants underwent the fMRI scanning procedure again and filled out the questionnaires.

The experiment was conducted in late summer/fall 2019 during daylight, between 10:00 a.m. and 5:00 p.m. The flowchart of the study procedure is shown in Fig. 1. Upon arrival, participants signed the informed consent, filled out the questionnaires, and performed a working memory task. Subsequently, the participants underwent an fMRI scanning procedure that included questions on rumination [23], the Fearful Faces Task (FFT) [24], and the Montreal Imaging Stress Task (MIST) [25]. The MIST was administered in order to induce social stress, since the SRT [13] hypothesizes that nature’s restorative potential is most evident when the individual is in a stressed state. The order of the FFT and MIST was counterbalanced between subjects, however, the order was the same within subjects, at pretest and posttest.

**Fig. 2:**
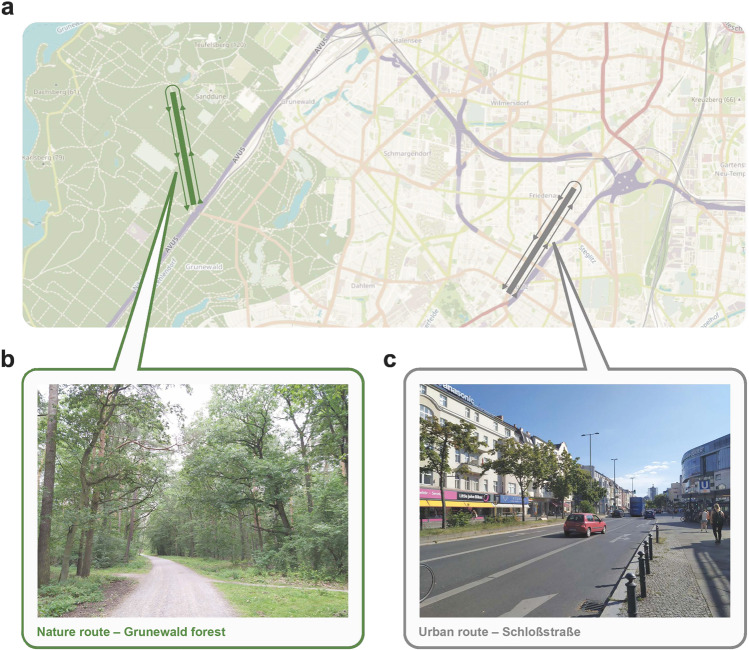
Location of the nature and urban walk. **a** GPS data of two participants during the walk in the natural environment (Berlin, Grunewald) and the urban environment (Berlin, Schloßstraße) displayed on the OpenStreetMap (https://www.openstreetmap.org). **b** Sample picture of the walk in the natural environment. **c** Sample picture of the walk in the urban environment.

After the scanning session, participants were randomly assigned to a 60-minute walk in either a natural or urban environment (Fig. 2). Even though the definition and also the dichotomy of ‘natural’ and ‘urban’ environment has been an object of debate [26], the ‘natural environment’ we refer to is an urban forest, the largest green area in the city of Berlin (Grunewald forest; Fig. 2b), whereas the ‘urban environment’ refers to a busy street in one of the city centers in Berlin with shopping malls (Schloßstraße; Fig. 2c). As recommended in the recent review [27], geographic locations of the walk and the landscape features of the environments are reported (see Supplementary information).

The participants were shown the exact walk route on a map (straight path) and they were collected at the lab and brought by taxi to the starting point of the walk. They carried a mobile phone that logged participants’ global positioning system (GPS) data during the walk, to ensure that they walked the intended route (Fig. 2a). During the walk, participants were equipped with an Empatica E4 (Empatica S.r.l, Milan, Italy), a wristband measuring electrodermal activity (EDA), heart rate variability (HRV), and heart rate, as physiological indicators of stress. Participants went on the walk alone and were instructed not to enter shops or use their mobile phones, to avoid potential distraction. They were given a bagged lunch that they could eat during the walk. After 30 min, as an alarm signal was generated by the phone, they turned around and continued the walk back to the starting point. Here they were picked up by a taxi and brought back to the lab.

After the walk the same fMRI scanning procedure was repeated, with one additional stress-inducing task, the Social-Evaluative Threat task (SET) [28], a modified version of the Trier Social Stress Test [29], meant to induce social stress and presented only after the walk, since we reasoned that the participants would not have believed the cover story twice (for detailed SET task procedure see Supplementary information). Additionally, the participants reported the level of restored attention after the walk via a questionnaire. Finally, the participants were debriefed and informed about the aim of the study. Within the scope of this article, we report on the fMRI results on the FFT and the MIST.

### Functional imaging paradigms

#### Fearful Faces Task (FFT)

**Fig. 3:**
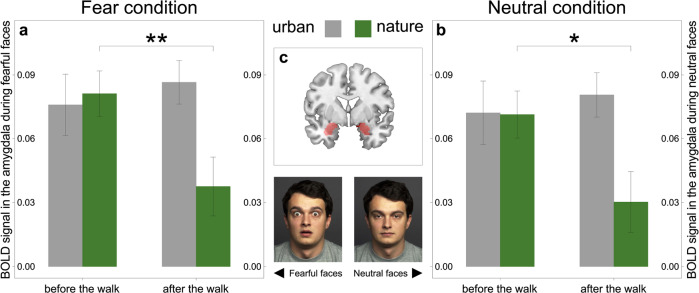
Bilateral amygdala activity during the Fearful Faces Task before and after the walk in the urban and in the natural environment. **a** a Bilateral amygdala activity while watching fearful faces (Fear condition) decreased after the walk in the natural environment. **b** Bilateral amygdala activity while watching neutral faces (Neutral condition) decreased after the walk in the natural environment. **c** Region of interest, the bilateral amygdala as defined in Automated Anatomic Labelling Atlas 2. Bottom: Stimuli in the Fearful Faces Task showing fearful facial expression, within the Fear condition (left) and neutral facial expression within the Neutral condition (right). Note: BOLD stands for Blood-Oxygen Level-Dependent; Significant differences are indicated with asterisks (*P < 0.05; **P < 0.01); error bars represent one standard error of the mean.

An adapted version of the Fearful Faces Task (FFT) [24] was used, designed to measure amygdala activity during fearful and neutral facial expressions. While in the MRI scanner, participants were presented with stimuli, consisting of 15 male and 15 female faces, each depicting fearful (Fear condition; Fig. 3 bottom left) or neutral facial expression (Neutral condition; Fig. 3 bottom right). Both fearful and neutral facial expressions were shown either for 1000 ms (unmasked stimuli) or for 17 ms followed by a mask with neutral facial expression presented for 983 ms (masked stimuli). Since the amygdala has been shown to respond to masked stimuli even when most of the participants were not aware of their presence [30–32], we used masked stimuli in order to exploratorily examine whether the degree of conscious perception had an effect on amygdala activity. However, we did not have the time to perform a perceptual control test and therefore we have no proof that the masked stimuli were actually processed outside of the participants’ awareness.

We used the set of 60 stimuli from the FACES database by the Max Planck Institute for Human Development in Berlin [33], consisting of face photographs on a gray background, matched on size and luminance. We used the FACES database because it provides a large set of validated high-resolution photographs with natural facial expressions that vary by gender, age, and emotion. The fMRI paradigm consisted of 22 blocks with 6 pictures interleaved with a 200 ms break between pictures. Each block was followed by a white fixation cross presented for 9 s. In order to monitor the participants’ attention, the fixation cross was red on two occasions, and participants were instructed to press the button on the response box as soon as they would see the red cross on the screen. The order of the stimuli was randomized within 10 versions of the FFT, and the task version was introduced in the fMRI data analysis as a covariate. The whole task sequence lasted 8 minutes and 28 s. The task was presented via a projector and mirror system and the participants answered using a response box. The FFT was presented using software Presentation (version: 19.0) and the code for the task used in this study is openly available at https://osf.io/5m2qv.

#### Montreal Imaging Stress Task (MIST)

The Montreal Imaging Stress Task (MIST) [25] is a computerized fMRI-adapted paradigm, based on the Trier Social Stress Test [29], with an aim to induce social stress, in which participants solve mental arithmetic tasks with a time limit designed to be just beyond the participant’s cognitive capacities. The MIST consisted of three different conditions: Experimental, Control, and Rest (Supplementary Fig. 1).

In the Experimental condition, the information about individual performance and a fake-average performance of all participants was graphically presented after each response. This fake-average performance was consistently considerably better than the individual performance in order to induce social stress. In the Control condition, the mental arithmetic tasks had the same level of difficulty as in the Experimental condition, but the participant’s performance as well as the fake-average performance of all participants was not displayed and there was no time limit for solving the task. In the Rest condition, treated as a baseline, no task was displayed and the participants were asked to simply passively look at the screen [25]. For detailed MIST procedure see Supplementary information.

### Magnetic Resonance Imaging

#### Data acquisition

All images were acquired on a Siemens Tim Trio 3 T scanner (Erlangen, Germany) using a 32-channel head coil. The T1-weighted images were obtained using a three-dimensional T1-weighted magnetization prepared gradient-echo sequence (MPRAGE; repetition time (TR) = 2500 ms; echo time (TE) = 4.77 ms; TI = 1100 ms, acquisition matrix = 256 × 256 × 192, flip angle = 7°; 1 x 1 x 1 mm^3^ voxel size). Whole brain functional images were collected using a T2*-weighted echo-planar imaging (EPI) sequence sensitive to BOLD contrast (TR = 2000 ms, TE = 30 ms, acquisition matrix = 216 × 216 × 129, flip angle = 80°, slice thickness = 3.0 mm, distance factor = 20%, FOV = 216 mm, 3 × 3 × 3 mm^3^ voxel size, 36 axial slices, using GRAPPA).

#### Data preprocessing

Functional imaging data were preprocessed and analyzed using Statistical Parametric Mapping software (SPM12; https://www.fil.ion.ucl.ac.uk/spm/software/spm12/). EPIs were corrected for slice timing and head motion and transformed into the stereotactic normalized standard space of the Montreal Neuroimaging Institute (MNI) using the unified segmentation algorithm. Finally, spatial smoothing with a 6-mm full width at half-maximum (FWHM) Gaussian kernel was performed. The voxel size was not changed during preprocessing but kept in the original acquisition dimension (3 × 3 × 3 mm^3^).

#### Data analysis

At the first level analysis of the FFT estimates of functional activation during conditions (unmasked Fear, unmasked Neutral, masked Fear, masked Neutral, Response) were obtained using an event-related paradigm. A high-pass filter (cut-off 128 s) was applied. Subsequently, a whole brain analysis was performed, using flexible factorial design with a focus on the interaction of environment (urban vs. natural) and time (before vs. after the walk). Both interaction contrasts were analyzed (Fear > Neutral and Neutral > Fear), using family-wise error (FEW) correction with a threshold at *P* < 0.05, and no significant clusters survived. Additionally, in order to perform a whole brain analysis with less rigorous threshold, the contrasts were thresholded at *P* < 0.001, uncorrected while controlling for multiple testing on the cluster level using 3DClutSim in AFNI (Analysis of Functional Neuroimages) [34] and again no significant clusters survived.

We then used a ROI-based approach, based on our a priori hypothesis, focusing on ROI amygdala, ACC (both derived from the Automated Anatomic Labelling atlas 2 [35], https://www.gin.cnrs.fr/en/tools/aal/), and dlPFC (left and right frontal superior gyrus), derived from the SPM Anatomy Toolbox [36], using WFU PickAtlas (https://www.nitrc.org/projects/wfu_pickatlas). Volume of the bilateral amygdala was 3744 mm^3^, dlPFC volume was 79,968 mm^3^, and ACC volume was 21,704 mm^3^. We extracted mean BOLD signal from a time window of 4–6 s after stimulus onset across all voxels within each ROI using a Matlab script based on the marsbar toolbox (version 0.44 [37]). We reasoned that the intervention, namely a one-hour walk, would globally affect the stress level and therewith stress-related brain activity, not only when contrasting the Fear > Neutral condition. To test this, we examined activity of each ROI (bilateral amygdala, dlPFC, and ACC) in Fear and Neutral condition separately. Since the results in both conditions were similar, we also examined pooled ROI activity of Fear and Neutral condition. We averaged data from unmasked and masked stimuli, because the results were similar.

We conducted a two-way mixed ANOVA with environment as a between-subject factor (urban vs. natural) and time as a within-subject factor (before vs. after the walk), in the Fear and Neutral condition separately, and also in the ROI pooled activity of Fear and Neutral conditions, while focusing on environment-by-time interaction. Two-tailed post-hoc *t*-tests were performed within the urban and the natural environment to examine the differences in ROI activity before and after the walk in each environment as well as separately within Fear and Neutral conditions, and the pooled activity of the latter conditions. Additionally, the amygdala subregions (centromedial and laterobasal amygdala) were derived from an atlas of the SPM Anatomy Toolbox [36] and the two-way mixed ANOVA was performed in the same ways as described above.

At the first level analysis of the MIST we obtained estimates of functional activation during the three conditions within a block-design paradigm (Experimental, Control, and Rest) and applied a high-pass filter (cut-off 520 s). We first performed a whole brain analysis, using flexible factorial model and focusing on the interaction of environment (urban vs. natural) and time (before vs. after the walk). Both interaction contrasts (Exp > Cont and Cont > Exp) were analyzed, using family-wise error correction with a threshold at *P* < 0.05 and no significant clusters survived. Subsequently, to present a more lenient thresholding, the contrasts were thresholded at *P* < 0.001, uncorrected while controlling for multiple testing on the cluster level using 3DClustSim in AFNI [34]. Significant clusters within the Experimental > Control contrast are shown in the Supplementary Table 2. No significant clusters survived within the Control > Experimental contrast.

To analyze ROI activity within the MIST, we extracted the beta values within each ROI separately for the contrasts Experimental > Rest and Control > Rest, in order to obtain the beta values in the Experimental and Control condition relative to baseline (Rest condition). Subsequently, a 2 x 2 x 2 mixed ANOVA was conducted with condition (Experimental vs. Control) and environment as a between-subject factor (urban vs. natural) and time as a within-subject factor (before vs. after the walk) for the amygdala activity, also focusing on environment-by-time interaction. Additionally, and in accordance with how the FFT data was analyzed, post-hoc *t*-tests were conducted with pooled amygdala activity of the Experimental and Control condition as a dependent variable in order to examine if the environment-by-time interaction was driven by a change in the amygdala activity after the walk in the urban or in the natural environment.

Behavioural data and Physiological data are reported in the Supplementary information.

## Results

As hypothesized, we observed a significant environment-by-time interaction in bilateral amygdala in the Fear [*F*(1,61) = 6.11, *P* = 0.016, η^2^_g_ = 0.04; Fig. 3a] as well as in the Neutral condition [*F*(1,61) = 4.86, *P* = 0.031, η^2^_g_ = 0.03; Fig. 3b]. Moreover, a significant environment-by-time interaction was likewise observed when bilateral amygdala activity within both Fear and Neutral conditions was pooled [*F*(1,61) = 5.81, *P* = 0.019, η^2^_g_ = 0.04]. There was no significant time-by-environment interaction neither in ACC or dlPFC in the FFT in the Fear condition (Supplementary Table 3), Neutral condition (Supplementary Table 4), nor in the pooled activity of the Fear and Neutral conditions (Supplementary Table 5).

To investigate whether the environment-by-time interaction in amygdala activity was mostly driven by an increase in the urban environment or by a decrease in the natural environment, we conducted follow-up *t*-tests. The two-tailed paired post-hoc *t*-tests for pooled activity during the Fear and Neutral condition revealed that amygdala activity was stable in the urban environment [*t*(30) = −0.67, *P* = 0.506], whereas there was a significant decrease in amygdala activity after the walk in nature [*t*(31) = 2.62, *P* = 0.014]. A two-tailed paired post-hoc *t*-test also showed a decrease in amygdala activity after the walk in natural environment when tested separately within the Fear [*t*(31) = 2.77, *P* = 0.009; Fig. 3a] and the Neutral condition [*t*(31) = 2.37, *P* = 0.024; Fig. 3b]. Therefore, the environment-by-time interaction was driven by a significant decrease in amygdala activity after the walk in nature (Fig. 3). Additionally, we observed that the interaction in amygdala activation was lateralized and mostly driven by the activity in the right amygdala [*F*(1,61) = 7.00, *P* = 0.010, η^2^_g_ = 0.04].

Interestingly, the analysis of bilateral amygdala activity only during masked stimuli as well revealed a significant environment-by-time interaction [*F*(1,61) = 5.58, *P* = 0.021, η^2^_g_ = 0.03], showing a decrease after the exposure to the natural environment [*t*(31) = 2.65, *P* = 0.012].

Exploratorily, we tested different subregions of the amygdala separately and observed a significant environment-by-time interaction in the basolateral amygdala [*F*(1,61) = 5.17, *P* = 0.026, η^2^_g_ = 0.03; Supplementary Fig. 2], likewise driven by a decrease in its activity after the walk in nature [*t*(31) = 1.98, *P* = 0.057].

As hypothesized and in the same direction as in the FFT, we observed a significant environment-by-time interaction in amygdala activity in pooled Experimental and Control condition in the MIST [*F*(1,61) = 5.07, *P* = 0.028, η^2^_g_ = 0.02; Supplementary Fig. 3]. Likewise in the FFT, two-tailed paired post-hoc *t*-tests within the MIST revealed that the interaction was driven by a decrease in amygdala activity after the walk in nature [*t*(31) = 1.88, *P* = 0.070], whereas amygdala activity remained stable after the walk in the urban environment [*t*(30) = −1.28, *P* = 0.211]. In the MIST, as in the FFT, there was no time-by-environment interaction in ACC or dlPFC (Supplementary Table 6).

There was no significant environment-by-time interaction in self-report measures or in the cognitive task (Supplementary Tables 7, 8) nor in physiological indicators of stress (Supplementary Table 9). However, as predicted, perceived restorativeness was higher after the nature walk than after the urban walk [*Z* = − 3.85, *P* < 0.001, *r* = 0.49; Supplementary Fig. 4 and Supplementary Table 10]. Moreover, participants who went for a walk in nature reported that they enjoyed the walk more [*Mdn* = 92, *IQR* = 20.5], compared to the participants who went for an urban walk [*Mdn* = 70, *IQR* = 40.5, *Z* = − 2.87, *P* = 0.004, *r* = 0.37].

## Discussion

Living in an urban environment has been associated with mental health problems, like anxiety disorders, depression, and schizophrenia, with urban upbringing being the most important environmental factor for developing schizophrenia [3, 4]. To investigate causal effects of urban and natural environments on the brain, we conducted an intervention study that examined changes in stress-related brain regions after a one-hour walk in an urban vs. natural environment. Furthermore, we aimed to explore whether stress-relief after exposure to nature is a result of the natural environment itself or of the mere absence of disadvantageous urban effects.

In line with our hypothesis, we observed that amygdala activity decreased after the walk in nature, whereas it remained the same after the walk in the urban environment. We interpret this as evidence showing that nature is indeed able to restore individuals from stress, and as a lack of evidence that the administered urban exposure additionally heightens amygdala activity.

We observed a decrease in amygdala activity after the walk in nature not only during fearful, but also during neutral faces in the FFT. The bilateral amygdala has been shown to respond to both fearful and neutral faces [38], although it is prominently reported that subtracting brain activity during neutral faces from that during fearful faces results in amygdala activity [24, 39, 40]. We speculate that the effect of exposure to nature was rather a general effect that affected the amygdala by increasing its threshold for activation, consequently leading to an interaction effect during both fearful and neutral faces.

Furthermore, we found that amygdala activity during masked stimuli showed the same effect as during unmasked stimuli, namely, it decreased after the walk in nature, whereas it remained stable after the walk in the urban environment. These results are in accordance with previous evidence showing that the amygdala can be activated in response to masked stimuli that participants were not aware of, in absence of cortical processing [30, 31] and suggest that the beneficial effect of nature exposure on stress may occur outside of our awareness.

Interestingly, we observed that the environment-by-time interaction effect was mostly driven by the activity in right amygdala, which is in line with the previous study showing lower amygdala activity in rural compared to urban dwellers, also lateralized to the right amygdala [21]. Exploratorily, we examined amygdala subregions separately and found an environment-by-time interaction (the activity remaining stable after the urban walk, whereas descriptively decreasing after the nature walk) in the basolateral amygdala activity, a subregion that has previously been reported in the context of fear conditioning [41] and to be activated during anxiety [42].

As predicted, and in line with the results on the FFT, we observed a significant environment-by-time interaction in amygdala activity also in the social stress task, the MIST, with amygdala activity remaining stable after the walk in the urban environment and descriptively decreasing after the walk in nature. These results indicate that predicted effects of walks in natural environments on stress-related brain regions occur also under conditions of social stress. The same pattern of amygdala activity after the exposure to the natural environment observed in both tasks, the FFT and the MIST, suggests that a one-hour walk in nature may have had a global beneficial effect on amygdala activity resulting in increasing amygdala’s threshold for activation, regardless of the task at hand. Since environment-by-time interaction was not observed in the ACC or dlPFC neither in the FFT nor in the MIST, the data imply that the amygdala may be a key stress-related brain region where the environment has an effect.

A possible explanation for why there was no observed change in behavioural measures after the walk may lie in the fact that the posttest questionnaires referred to mood and stress experienced during the previous hour, when participants were undergoing the fMRI stress-inducing paradigm. Therefore, we believe that the questionnaires were not able to capture the effect of the walk, but rather the effect of the stress-inducing paradigm. In future studies, behavioural measures should be administered in a short form as soon as participants come back from the walk, in order to capture the effect of the walk within both the questionnaires and the fMRI paradigm.

However, perceived restorativeness, referring to restored attention during the walk, was reported to be higher after the walk in nature than after the urban walk, which is in line with the ART [14] and previous studies [8] showing that natural environments have restorative benefits on attention. Moreover, participants who went for a nature walk enjoyed the walk more than those who went for a walk in the urban environment, a finding that is consistent with participants’ higher restorativeness as well as lower amygdala activity after the walk in nature.

According to ART, natural environments restore cognition, whereas within the SRT framework, nature-induced restoration is related to recovery from stress. Even though ART and SRT are complementary theoretical frameworks [8, 9], in the context of this study, ART would emphasize restored cognition and therefore effects in cognitive brain areas, whereas SRT would focus rather on the importance of stress-related brain areas. Since the results show a decrease in stress-related brain areas (bilateral amygdala) after the walk in nature, and no change in cognition-related brain areas (dlPFC and ACC), the brain data of the present study are more strongly in line with SRT.

To the best of our knowledge, this is the first study to demonstrate the causal effects of acute exposure to a natural vs. urban environment on stress-related brain regions, disentangling positive effects of nature from negative effects of city. We demonstrated that amygdala activation decreased during a stress task after nature exposure, whereas it remained stable after urban exposure. This strongly argues in favor of the salutogenic effects of nature as opposed to urban exposure causing additional stress.

The results presented may reveal the mechanism behind the long-term effects of the environment on stress-related brain regions. The decrease in amygdala activity as a result of acute exposure to nature might be a mechanism explaining lower amygdala activity during stress in rural dwellers [21] and higher structural amygdala integrity in citizens who live close to urban forests [43]. Repeated exposures to nature may beneficially affect amygdala by increasing its threshold for activation, resulting in lower amygdala activity during stress and higher amygdala integrity in habitants who live close to natural environments.

Detrimental effects of urban environments related to higher schizophrenia incidence in cities, like social stress, might be attenuated with exposure to natural environments through decreased stress-related amygdala activation. Since schizophrenia has been related to urban upbringing [4] and amygdala alterations [44], spending time in urban nature (green prescription) and consequently decreasing amygdala engagement may buffer the disadvantageous impact of the city and serve as a preventive measure against developing schizophrenia. Higher association between urbanicity and schizophrenia in recent birth cohorts and rapidly increasing urbanization suggest that effects of urban environment may increase in the future [4], underlining the responsibility of urban planning to focus on modifying current and future cities to provide accessible green spaces in order to improve citizens’ mental health.

One of the limitations of the study is the lack of evidence that the masked facial stimuli in the FFT were not consciously perceived, since we have not explicitly tested for this. We would recommend that future studies perform a perceptual control task in order to ensure that participants did not consciously perceive the masked stimuli. Secondly, it is not clear which aspects of nature are driving the effect of the decrease in amygdala activation after exposure to natural environment. Therefore, future studies should aim to pinpoint specific features of nature that are beneficial and drive the decrease in amygdala activity (e.g., green color, sound, odors, terpenes etc.) in order to understand why nature induces restorative processes and, consequently, to make nature-based therapy more efficient. Thirdly, even though the Grunewald forest path where the participants went for a walk is isolated from the city, participants might have seen other people engaged in spare time activities, such as walking or exercising, which could have contributed to higher relaxation and lower amygdala activity after the walk in nature compared to the urban walk. Hence, future studies should control for number of people encountered during the walk as well as for their affective state, since this may be different in natural and urban environments. Fourthly, different natural environments may have different effects on participants (e.g., a forest could elicit fear instead of relaxation [45] and walking in a tended forest may have a more positive impact on well-being than walking in a wild forest [46]). Therefore, future studies should examine changes in stress-related brain regions after exposure to different types of natural environments, for example to an urban park or a botanical garden. Finally, since the assignment of meaning to nature likely differs across cultures [47, 48], future research should try to include participants from different cultural backgrounds in order to examine whether the beneficial effects of nature on stress-related brain regions differ across cultures.

To conclude, our results demonstrate that exposure to nature for one hour decreases amygdala activity and can have salutogenic effects on brain regions related to stress. This suggests that going for a walk in nature may buffer detrimental effects of urban environment on stress-related brain regions, and in turn potentially act as a preventive measure against developing a mental disorder.

## Supplementary information

Supplementary infromation

## Data Availability

The data supporting the findings of this study are publicly available at https://osf.io/5m2qv/.

## Code availability

The code associated with the data analysis within this study as well as tasks-related code is publicly available at https://osf.io/5m2qv
