# Supplementary material for: How nature nurtures: Amygdala activity decreases as the result of a one-hour walk in nature
Source: Mol Psychiatry. 2022 Sep 5;27(11):4446–52. doi: 10.1038/s41380-022-01720-6 (PMC9734043; doi:10.1038/s41380-022-01720-6)
Supplement: Supplementary file 1 — Supplementary infromation [file 41380_2022_1720_MOESM1_ESM.pdf]

Supplementary information

METHODS

Location of the walks

As recommended in a recent review[1], we report the geographic location of the walks as well as the landscape features. The walk in nature took place in Grunewald forest in Berlin, Germany (Fig. 2b), close to the MRI laboratory (6.7 km, reached within approximately 15 minutes by taxi). The participants walked along Teltower Weg, a path starting at a crossroads between Königsweg and Teltower Weg, spanning towards the north of Grunewald. At over 3000 hectares, Grunewald forest is the largest green area in the city of Berlin. The vegetation of the area is mainly composed of conifers and Betulaceae. Some areas of Grunewald forest are nature reserves and therefore forbidden to visit to protect local fauna, especially amphibians and birds. Apart from the river Havel which forms small islands and a peninsula, Grunewald forest is rich in lakes and ponds, however, the walking route did not pass any water. There are no built structures nor was there any traffic noise during the walking route the participants undertook. Since Grunewald is a recreative forest, it has many paths that people mostly use for walking, jogging, or riding a bicycle.

The urban walk took place in Schloßstraße, a busy street in Berlin-Steglitz (Fig. 2c), close to the MRI laboratory (2.1 km, reachable in approximately 10 minutes by taxi), which consists of two to four traffic lanes. Schloßstraße is one of Berlin's shopping areas, with three shopping malls and three subway stations. Participants were dropped off by the taxi at Schloßstraße 84 and walked northeast, towards Rheinstraße. The participants walked along the sidewalk and could see other people, traffic, buildings, shopping malls, and smaller shops. In Schloßstraße, however, as in most of Berlin's streets, there were also trees, mostly on the sidewalk and on the traffic dividers.

Montreal Imaging Stress Task (MIST)

The Montreal Imaging Stress Task (MIST), a computerized fMRI-adapted task[2], was presented via same projector and mirror system as the FFT and the participants also answered using a response box. In the beginning of the task there was a training session in which the participants' ability to perform mental arithmetic was evaluated, without time limit and a progress bar, to set a default time limit in the Experimental condition.

In the Experimental condition (Suppl. Fig. 1, left), the MIST program reduced the time limit to 10% less than the participant's average time after three correctly solved tasks. When the participants responded incorrectly on three consecutive tasks, the program increased the time limit for the following tasks by 10%. This staircase procedure in the Experimental condition leads to a range of about 20% to 45% of correct answers[2]. In the Experimental condition the information about individual performance and a fake-average performance of all participants was presented after each response with arrows on a performance bar above arithmetic tasks in order to induce social stress. The mathematical arithmetic tasks were designed so that only one digit between 0 and 9 was the correct response. To respond, participants selected a digit on the rotary dial from 0 to 9 by pressing the left or the right button on the button box to highlight the neighboring left or right number until they reached the number they intended to respond with; in that case the middle button was used to confirm the answer. The participant's answer was compared with the correct answer for the arithmetic task and the feedback "Correct" or "Incorrect" was shown in the feedback field. If the time for the arithmetic task ran out, the feedback "Time out" was displayed.

In the Control condition (Suppl. Fig. 1, middle), the mental arithmetic tasks were as difficult as in the Experimental condition. However, the tasks had no time limit and the performance bar comparing the participant's performance and the fake-average performance was not displayed. The feedback for each task was also displayed, but since there was no time limit, average correct performance in the Control condition is around 80% to 90%[2].

Rest condition (Suppl. Fig. 1, right), the participants saw the rotary dial and empty fields for arithmetic tasks and the feedback, but no task was displayed and the participants were asked to simply look at the screen.

The order of the three conditions was randomized within 6 versions of the MIST and the task sequence lasted 14 minutes and 8 seconds. The MIST was retrieved from https://www.millisecond.com, adapted to German language and presented by means of the software Inquisit (version: 5.0.14.0). The code for the MIST together with the stimuli used in this study is openly available at https://osf.io/5m2qv

Social-Evaluative Threat task (SET)

The Social-Evaluative Threat task (SET)[3] is a modified version of the Trier Social Stress Test[4]. While in the MRI scanner, after a baseline phase of two minutes, participants were instructed to prepare for two minutes a 7-minutes speech on the topic "Why am I a good friend?", that they would have to perform in the scanner while being audio- and video-recorded. After the stressor phase, during which

fMRI data were acquired, participants were informed that they were randomly selected not to give the speech. The SET task was introduced in order to induce social stress and was presented only at posttest in order to enhance the credibility of the paradigm. At the end of the experiment, during debriefing, participants were informed that no participant had to give the talk. Due to scope of this paper and SET data analysis that differs from the FFT and MIST analysis, the results on the SET task are not reported on here.

Behavioural data

Behavioural measures included questionnaires assessing mood (German version of Positive and Negative Affect Schedule, PANAS[5]), perceived stress during previous hour (adapted German version of Perceived Stress Scale, PSS[6]), rumination during previous hour (adapted Rumination subscale from German version of Rumination Reflection Questionnaire, RRQ[7]), and perceived restorativeness (German version of Perceived Restorativeness Scale, PRS[8]), in addition to a Digit Span Backwards (DSB) task assessing working memory[9]. All behavioural measures were administered at pretest and posttest, except for the PRS which assesses the perceived restorativeness of an environment and was, therefore, reasonable to use only after the walk. Additionally, participants filled out a sociodemographic questionnaire, reported on the weather during the walk as well as the overall pleasantness of the walk, and responded to a German version of the Connectedness to Nature questionnaire[10].

Questionnaires that were administered at pretest and posttest (PANAS, PSS, RRQ) and the DSB task were analyzed using a two-way ANOVA with time as a within-subject factor (before the walk vs. after the walk) and the environment as a between-subject factor (urban vs. natural environment). Due to technical problems, there were missing data for one participant on the DSB task, PANAS, and PSS. Behavioural data from one participant who dropped out at posttest were excluded. As preregistered, participants who scored below or above 2.5 standard deviations from the mean were treated as outliers and excluded from the analyses. Two outliers in the PANAS subscale Negative Affect and in the DSB task were detected and therefore excluded from further analysis. The final sample size for each behavioural measure are reported in the Supplementary Table 8.

PANAS subscale Positive Affect and the RRQ subscale Rumination met the normality assumption and were analyzed using the ezANOVA function from the R package ez[11]. However, since the Shapiro-Wilk normality test indicated that the data were not normally distributed across groups in the PANAS subscale Negative Affect, in the PSS as well as in the DSB, the data for these measures was analyzed with robust ANOVA using the R package WRS2[12]. The Shapiro-Wilk normality test

showed that the data normality assumption was not met in the PRS and the question about the pleasantness of the walk, therefore a two-tailed Wilcoxon rank sum test for independent samples was performed, with environment as the independent variable (urban vs. natural environment). The analyses were performed using R software (https://cran.r-project.org/src/base/).

Physiological data

High frequency power band (HF) of heart rate variability (HRV) from the blood volume pulse was selected as a measure of parasympathetic activity[13-16], whereas low frequency divided by high frequency power band (LF/HF) from HRV and heart rate (HR)[13, 14] were used as an index of sympathetic activity[15-21]. LF/HF ratio was computed based on the pre-processed values of LF and HF power band of HRV. HR values were taken directly form the wristband raw data. Electrodermal activity (EDA) signal was decomposed into its phasic and tonic components, and the phasic component was taken as an indicator of sympathetic activity[22, 23]. EDA and blood volume pulse were first preprocessed using the pyPhysio library[24] in Python (https://www.python.org/).

A fixed-length windowing approach was employed[25], resulting in signal indicators' average for each minute. Since the walk lasted up to 60 minutes, the first 54 minutes of the walk were taken into analysis and split into three 18-minute time windows. In order to examine if there was a significant difference in physiological indicators of stress during the walk in the urban vs. natural environment, independent samples t-tests were performed on EDA phasic component, HR, HF power band of HRV and LF/HF ratio of HRV. Physiological indicators of stress were compared between the urban and the natural environment for each of the three 18-minute time windows. Data in time-window 2 and 3 of LF/HF ratio was non-normally distributed, thus a Wilcoxon signed rank test was performed. Since the physiological data of 11 participants were not recorded due to technical difficulties, 52 participants were included in the physiological data analysis. The EDA signal of one participant was too low to be detected after signal processing in Python, resulting in a subsample of 51 participants in the EDA phasic component analysis.

Supplementary Figures

Supplementary Fig. 1: Montreal Imaging Stress Task (MIST) in each of the conditions.

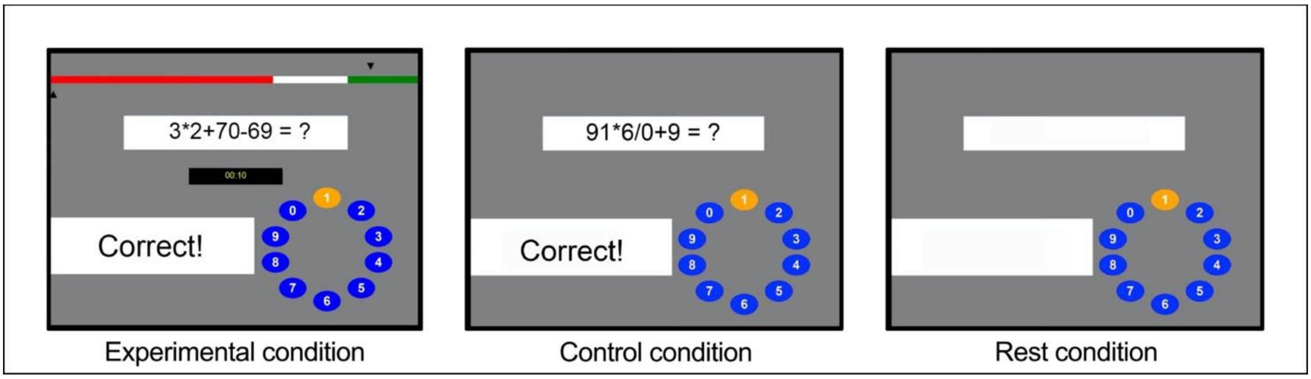

Supplementary Fig.1. Graphical user interface of the Montreal Imaging Stress Task (MIST) in each of the conditions. **Left:** Experimental condition with a bar representing participant's performance (bottom arrow) and fake-average performance (top arrow), the mental arithmetic task, the field showing remaining time for the task, the feedback field and the rotary dial for the response submission; **Middle:** Control condition with the mental arithmetic task, the feedback field and the rotary dial; **Right:** Rest condition with the rotary dial and without mental arithmetic task and feedback.

Supplementary Fig. 2: Basolateral amygdala activity during the Fearful Faces Task before and after the walk in the urban and in the natural environment.

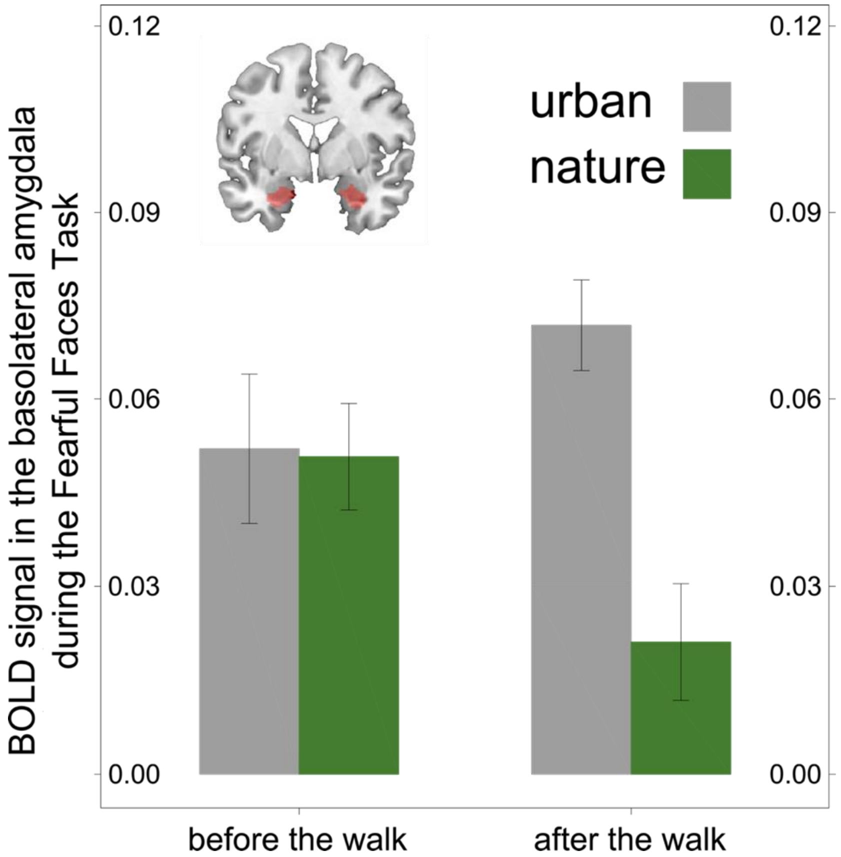

Supplementary Fig. 2. Bilateral basolateral amygdala activity during the Fearful Faces Task (pooled activity during fearful faces and neutral faces) before and after the walk in the urban and in the natural environment. **Top left:** Basolateral amygdala, derived from the SPM Anatomy Toolbox[26].
Note: Error bars represent one standard error of the mean.

Supplementary Fig. 3: Amygdala activity during the Montreal Imaging Stress Task before and after the walk in the urban and in the natural environment.

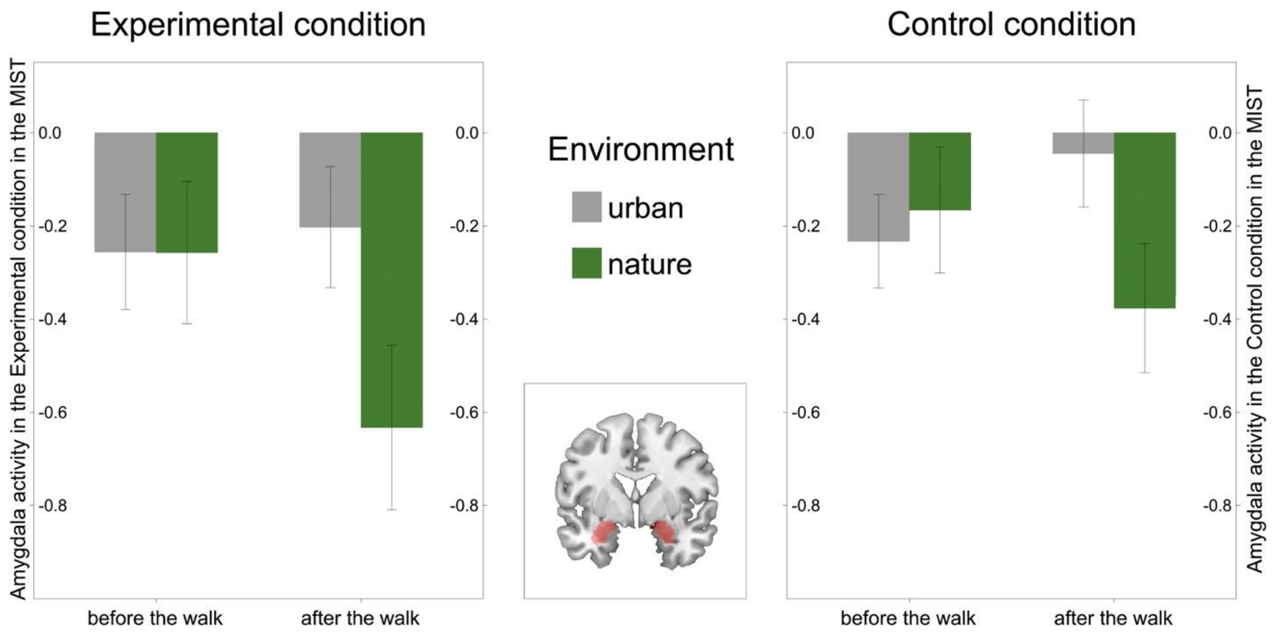

Supplementary Fig. 3. Bilateral amygdala activity during Montreal Imaging Stress Task before and after the walk in the urban and in the natural environment. **Left:** Amygdala activity (beta values) in the Experimental condition. **Right:** Amygdala activity (beta values) in the Control condition. **Middle:** Region of interest, the bilateral amygdala activity as defined in Automated Anatomic Labelling Atlas 2.
Note: Error bars represent one standard error of the mean.

Supplementary Fig. 4: Perceived restorativeness of the walk in the urban and in the natural environment.

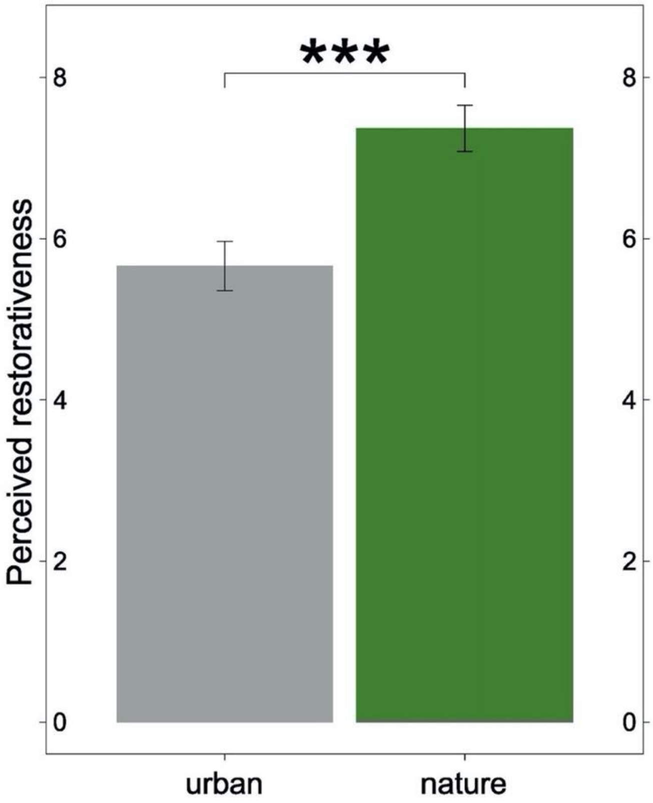

Supplementary Fig. 4. Score on Perceived Restorativeness Scale after the walk in the urban and in the natural environment.

Note: Error bars represent one standard error from the mean. Significant differences are indicated with asterisks (*** $P < 0.001$).

Supplementary Tables

Supplementary Table 1: Control variables values in the urban and in the natural environment.

Variable	Urban (n = 31)	Nature (n = 32)	χ2/ *t*	*df*	*P* value
Age (mean ± *SD*)	28.58 ± 7.41	25.87 ± 5.52	1.64	55.40	0.107
Sex: female (%)	45	47	0.02	1	0.891
Country of origin: Germany (%)	84	91	0.65	1	0.421
Upbringing: grew up in a city (%)	58	66	0.38	1	0.537
Income: under 1250 euros (%)	71	87.5	2.63	1	0.105
Occupation: students (%)	65	75	0.82	1	0.365
Education: high school diploma (%)	90	91	0.002	1	0.967
Day time: afternoon sessions (%)	45	47	0.02	1	0.891
Air temperature[a] (mean ± *SD*)	16.05 ± 5.12	14.72 ± 5.37	1.00	60.99	0.319
Humidity (%)	68.26 ± 17.82	72.62 ± 19.06	−0.94	60.93	0.351
Cloudiness[b] (mean ± *SD*)	5.71 ± 1.99	6.25 ± 1.85	−1.12	60.35	0.269
Sunny: minutes during 60-minute walk (mean ± *SD*)	30.65 ± 22.62	20.38 ± 22.10	1.82	60.81	0.073
Rainy: walks in the rain (%)	29	31	0.88	1	0.348

Note: Weather values refer to 60 minutes of the walking time; weather data were obtained from the German Meteorological Service (https://www.dwd.de/); *df* = degrees of freedom; *SD* = standard deviation; *n* = 63

[a]Degrees Celsius (°C)

[b]Amount of cloudiness on an 8-point scale

Supplementary Table 2: Coordinates and anatomical regions significantly activated in the Experimental > Control condition in the Montreal Imaging Stress Task (MIST)

Anatomical region	*t* (peak level)	Voxels	x	y	z
left insula	3.92	129	-15	26	17
right postcentral gyrus	4.37	75	21	-34	62
left superior temporal gyrus	4.01	66	-48	-37	11
left inferior parietal lobule	3.80	40	-39	-25	29

Note: Statistical threshold at $P < 0.001$, uncorrected, while controlling for multiple testing on the cluster level using 3DClutSim within AFNI (Analysis of Functional Neuroimages)[27], k > 34. Anatomical regions were identified using xjView toolbox (https://www.alivelearn.net/xjview).

Descriptive statistics and ANOVA results for brain regions of interest activity in the Fearful Faces Task

Supplementary Table 3: Descriptive statistics and ANOVA results for brain regions of interest activity during fearful faces (Fear condition) in the Fearful Faces Task.

**ROI**	**Mean ± *SD***				***df***	***F***	***P* value**	**η^2_g**
	**Urban (*n* = 31)**		**Nature (*n* = 32)**					
	**pretest**	**posttest**	**pretest**	**posttest**				
Bilateral amygdala	0.08 ± 0.08	0.09 ± 0.06	0.08 ± 0.06	0.04 ± 0.08	61	6.11	0.016*	.04
ACC	-0.01 ± 0.06	0.00 ± 0.05	-0.01 ± 0.05	-0.02 ± 0.05	61	1.51	0.224	.01
dlPFC	-0.04 ± 0.05	-0.02 ± 0.04	-0.02 ± 0.05	-0.03 ± 0.04	61	2.16	0.147	.02

Note: Descriptive statistics and a two-way mixed ANOVA interaction effect of factors environment (urban vs. natural) and time (pretest vs. posttest) for each of the region of interest during fearful faces (Fear condition); ROI = region of interest; *SD* = standard deviation; *df* = degrees of freedom; η^2_g= generalized eta-squared effect size; ACC = anterior cingulate cortex; dlPFC = dorsolateral prefrontal cortex.
*$P < 0.05$

Supplementary Table 4: Descriptive statistics and ANOVA results for brain regions of interest activity during neutral faces (Neutral condition) in the Fearful Faces Task.

**ROI**	**Mean ± *SD***				***df***	***F***	***P* value**	**η^2_g**
	**Urban (*n* = 31)**		**Nature (*n* = 32)**					
	**pretest**	**posttest**	**pretest**	**posttest**				
Bilateral amygdala	0.07 ± 0.08	0.08 ± 0.06	0.07 ± 0.06	0.03 ± 0.08	61	4.86	0.031*	.03
ACC	-0.01 ± 0.06	-0.01 ± 0.06	0.00 ± 0.06	-0.02 ± 0.06	61	0.81	0.371	.01
dlPFC	-0.03 ± 0.05	-0.02 ± 0.05	-0.02 ± 0.04	-0.03 ± 0.04	61	0.57	0.455	.00

Note: Descriptive statistics and a two-way mixed ANOVA interaction effect of factors environment (urban vs. natural) and time (pretest vs. posttest) for each of the region of interest during neutral faces (Neutral condition); ROI = region of interest; *SD* = standard deviation; *df* = degrees of freedom; η^2_g= generalized eta-squared effect size; ACC = anterior cingulate cortex; dlPFC = dorsolateral prefrontal cortex.
*$P < 0.05$

Supplementary Table 5: Descriptive statistics and ANOVA results for pooled activity of brain regions of interest during fearful and neutral faces (Fear and Neutral condition) in the Fearful Faces Task.

ROI	Mean ± *SD*				*df*	*F*	*P* value	η^2_g
	Urban (*n* = 31)		Nature (*n* = 32)					
	pretest	posttest	pretest	posttest				
Bilateral amygdala	0.07 ± 0.08	0.08 ± 0.06	0.08 ± 0.06	0.03 ± 0.08	61	5.81	0.019*	.04
ACC	-0.01 ± 0.06	0.00 ± 0.06	0.00 ± 0.05	-0.02 ± 0.05	61	1.18	0.282	.01
dlPFC	-0.03 ± 0.05	-0.02 ± 0.05	-0.02 ± 0.04	-0.03 ± 0.04	61	1.29	0.261	.01

Note: Descriptive statistics and a two-way mixed ANOVA interaction effect of factors environment (urban vs. natural) and time (pretest vs. posttest) for each of the region of interest during Fearful Faces Task (pooled activity during fearful faces and neutral faces); ROI = region of interest; *SD* = standard deviation; *df* = degrees of freedom; η^2_g= generalized eta-squared effect size; ACC = anterior cingulate cortex; dlPFC = dorsolateral prefrontal cortex.

*P < 0.05

Descriptive statistics and ANOVA results for brain regions of interest activity in the Montreal Imaging Stress Task (MIST)

Supplementary Table 6: Descriptive statistics and ANOVA results for brain regions of interest activity in the Montreal Imaging Stress Task (MIST).

ROI	Mean ± *SD*				*df*	*F*	*P* value	η^2_g
	Urban (n = 31)		Nature (n = 32)					
	pretest	posttest	pretest	posttest				
Bilateral amygdala	-0.24 ± 0.54	-0.12 ± 0.63	-0.21 ± 0.75	-0.50 ± 0.72	61	5.07	0.028*	.02
ACC	0.00 ± 0.45	0.08 ± 0.63	-0.10 ± 0.56	-0.36 ± 0.74	61	3.16	0.080	.02
dlPFC	-0.01 ± 0.41	0.00 ± 0.48	-0.02 ± 0.40	-0.21 ± 0.49	61	2.16	0.146	.01

Note: Descriptive statistics (pooled activity of Experimental and Control condition) and environment-by-time interaction effect within a 2x2x2 ANOVA of factors environment (urban vs. natural), time (pretest vs. posttest) and condition (Experimental vs. Control) for each of the region of interest in the Montreal Imaging Stress Task; ROI = region of interest; *SD* = standard deviation; *df* = degrees of freedom; η^2_g= generalized eta-squared effect size; ACC = anterior cingulate cortex; dlPFC = dorsolateral prefrontal cortex.
*$P < 0.05$

Supplementary Table 7: Descriptive statistics of behavioural variables.

	Urban		Nature	
**Variable**	**pretest**	**posttest**	**pretest**	**posttest**
Positive affect[a] (mean ± *SD*)	29.63 ± 5.86	26.80 ± 6.80	27.87 ± 4.53	24.42 ± 8.42
Negative affect[b] (median ± *IQR*)	12.00 ± 4.00	12.00 ± 4.00	12.00 ± 3.00	14.00 ± 7.00
Perceived stress[c] (median ± *IQR*)	16.00 ± 6.50	26.50 ± 8.50	17.00 ± 6.50	27.00 ± 13.00
Rumination[d] (mean ± *SD*)	36.65 ± 8.73	36.65 ± 9.98	42.03 ± 9.62	41.13 ± 9.82
Working memory[e] (median ± *IQR*)	9.00 ± 3.00	9.00 ± 2.50	8.00 ± 2.50	9.00 ± 3.00

Note: SD = standard deviation; *IQR* = interquartile range.

[a]Positive and Negative Affect Schedule (PANAS), subscale Positive Affect. Score ranging from 10 to 50.

[b]Positive and Negative Affect Schedule (PANAS), subscale Negative Affect. Score ranging from 10 to 50.

[c]Perceived Stress Scale. Score ranging from 10 to 50.

[d]Rumination Reflection Questionnaire, subscale Rumination. Score ranging from 12 to 60.

[e]Digit Span Backwards task. Score ranging from 0 to 14.

Supplementary Table 8: ANOVA results of behavioural data.

Variable	*n*	*df*	*F*	*P value*
Positive affect	61	59	0.13	0.719
Negative affect	59	30.47	1.50	0.231
Perceived stress	61	33.93	0.00	0.981
Rumination	62	60	1.42	0.237
Working memory	59	33.90	0.02	0.884

Note: Two-way mixed ANOVA interaction effect of factors environment (urban vs. natural) and time (pretest vs. posttest). The sample size is different because of different number of outliers and data not recorded due to technical problems; *n* = sample size; *df* = degrees of freedom.

Supplementary Table 9: T-test results of physiological indicators of stress.

Variable	Time window	Urban	Nature	*df*	*t*	*P* value
		**Mean ± *SD***				
EDA phasic		**(*n* = 26)**	**(*n* = 25)**			
	Time window 1	0.10 ± 0.16	0.10 ± 0.17	47.91	-0.00	0.99
	Time window 2	0.14 ± 0.14	0.15 ± 0.17	45.21	0.12	0.90
	Time window 3	0.29 ± 0.32	0.23 ± 0.21	42.63	-0.84	0.40
HR		**(*n* = 26)**	**(*n* = 26)**			
	Time window 1	69.82 ± 13.03	69.59 ± 16.47	47.48	-0.06	0.95
	Time window 2	79.18 ± 14.35	79.67 ± 20.18	45.14	0.10	0.92
	Time window 3	83.11 ± 14.35	82.98 ± 17.20	48.45	-0.03	0.98
HF power band of HRV		**(*n* = 26)**	**(*n*= 26)**			
	Time window 1	797.68 ± 73.49	794.31 ± 64.81	50.00	-0.18	0.86
	Time window 2	789.13 ± 66.51	799.85 ± 71.66	49.72	0.56	0.58
	Time window 3	785.05 ± 71.37	803.15 ± 92.97	46.87	0.79	0.44
LF/HF of HRV		**(*n* = 26)**	**(*n*= 26)**			
	Time window 1	0.76 ± 0.08	0.74 ±0.09	50.00	-0.56	0.58
		**Median ± *IQR***			***z***	***P* value**
	Time window 2	0.71 ± 0.09	0.72 ± 0.08		1.75	0.96
	Time window 3	0.73 ± 0.06	0.74 ± 0.12		0.39	0.65

Note: Results from the t-test analysis examining differences on physiological indicators of stress during the walk in the urban vs. natural environment; Data in time-window 2 and 3 of LF/HF ratio of HRV was non-normally distributed, thus a Wilcoxon signed rank test was performed. *SD* = standard deviation; *IQR* = interquartile range; *n* = sample size; *df* = degrees of freedom. EDA phasic = phasic component of electrodermal activity; HR = heart rate; HF power band of HRV = high frequency power band of heart rate variability; LF/HF of HRV= low frequency power band divided by high frequency power band of heart rate variability.

Supplementary Table 10: Descriptive statistics and results on Perceived restorativeness

	Median ± *IQR*					
	**Urban (n = 31)**	**Nature (n = 31)**	n	z	***P* value**	r
Perceived restorativeness[a]	5.58 ± 2.21	7.75 ± 1.50	62	−3.85	0.0001***	0.49

Note: Wilcoxon rank sum test examining differences on Perceived restorativeness after the walk in the urban vs. natural environment; Due to non-normal data distribution, Wilcoxon rank sum test was performed; *IQR* = interquartile range; n = sample size; r = effect size (calculated as absolute value of the z score divided by the square root of the sample size).

[a]Perceived restorativeness scale. Scale ranging from 1 to 10.

***$P < 0.001$

REFERENCES

1. Barnes, M.R., *et al.* Characterizing nature and participant experience in studies of nature exposure for positive mental health: An integrative review. *Frontiers in Psychology* **9**, 2617 (2019).
2. Dedovic, K., *et al.* The Montreal Imaging Stress Task: using functional imaging to investigate the effects of perceiving and processing psychosocial stress in the human brain. *Journal of Psychiatry and Neuroscience* **30**, 319 (2005).
3. Wager, T.D., *et al.* Brain mediators of cardiovascular responses to social threat: Part I: Reciprocal dorsal and ventral sub-regions of the medial prefrontal cortex and heart-rate reactivity. *NeuroImage* **47**, 821-835 (2009).
4. Kirschbaum, C., Pirke, K.-M. & Hellhammer, D.H. The 'Trier Social Stress Test'–a tool for investigating psychobiological stress responses in a laboratory setting. *Neuropsychobiology* **28**, 76-81 (1993).
5. Krohne, H., Egloff, B., Kohlmann, C. & Tausch, A. Investigations with a German version of the positive and negative affect schedule (PANAS). *Diagnostica* **42**, 139-156 (1996).
6. Klein, E.M., *et al.* The German version of the Perceived Stress Scale–psychometric characteristics in a representative German community sample. *BMC psychiatry* **16**, 1-10 (2016).
7. Elkhaouda, S. *Geschlechtsspezifische Faktoren in der Vorhersage der Depressivität bei Jugendlichen* (2010).
8. Schönbauer, R. Das Potenzial privater Gärten für die wahrgenommene Gesundheit. (uniwien, 2013).
9. Berman, M.G., Jonides, J. & Kaplan, S. The cognitive benefits of interacting with nature. *Psychol Sci* **19**, 1207-1212 (2008).
10. Cervinka, R., Zeidler, D., Karlegger, A. & Hefler, E. Connectedness with nature, well-being and time spent in nature. in *Program and Abstract of 8th Biennial Conference, Environmental Psychology, September, 6th–9th (S. 130). Lengrich: Pabst Science Publishers* (2009).
11. Lawrence, M.A. & Lawrence, M.M.A. Package 'ez'. *R package version* **4** (2016).
12. Mair, P. & Wilcox, R. Robust statistical methods in R using the WRS2 package. *Behavior research methods* **52**, 464-488 (2020).
13. Jo, H., Song, C. & Miyazaki, Y. Physiological Benefits of Viewing Nature: A Systematic Review of Indoor Experiments. *Int J Environ Res Public Health* **16**, 4739 (2019).
14. Lanki, T., *et al.* Acute effects of visits to urban green environments on cardiovascular physiology in women: A field experiment. *Environ Res* **159**, 176-185 (2017).
15. Lee, J., *et al.* Effect of forest bathing on physiological and psychological responses in young Japanese male subjects. *Public Health* **125**, 93-100 (2011).
16. Shaffer, F., McCraty, R. & Zerr, C.L. A healthy heart is not a metronome: an integrative review of the heart's anatomy and heart rate variability. *Frontiers in psychology* **5**, 1040 (2014).
17. Park, B.J., Tsunetsugu, Y., Kasetani, T., Kagawa, T. & Miyazaki, Y. The physiological effects of Shinrin-yoku (taking in the forest atmosphere or forest bathing): evidence from field experiments in 24 forests across Japan. *Environ Health Prev Med* **15**, 18-26 (2010).
18. Park, B.J., *et al.* Physiological effects of Shinrin-yoku (taking in the atmosphere of the forest) in a mixed forest in Shinano Town, Japan. *Scandinavian Journal of Forest Research* **23**, 278-283 (2008).
19. Ochiai, H., Song, C., Ikei, H., Imai, M. & Miyazaki, Y. Effects of visual stimulation with bonsai trees on adult male patients with spinal cord injury. *International journal of environmental research and public health* **14**, 1017 (2017).
20. Weise, F. & Heydenreich, F. Effects of modified respiratory rhythm on heart rate variability during active orthostatic load. *Biomedica Biochimica Acta* **48**, 549-556 (1989).

21. Goldstein, D.S., Bentho, O., Park, M.Y. & Sharabi, Y. Low-frequency power of heart rate variability is not a measure of cardiac sympathetic tone but may be a measure of modulation of cardiac autonomic outflows by baroreflexes. *Experimental physiology* **96**, 1255-1261 (2011).
22. Benedek, M. & Kaernbach, C. A continuous measure of phasic electrodermal activity. *J Neurosci Methods* **190**, 80-91 (2010).
23. Braithwaite, J.J., Watson, D.G., Jones, R. & Rowe, M. A guide for analysing electrodermal activity (EDA) & skin conductance responses (SCRs) for psychological experiments. *Psychophysiology* **49**, 1017-1034 (2013).
24. Bizzego, A., Battisti, A., Gabrieli, G., Esposito, G. & Furlanello, C. pyphysio: A physiological signal processing library for data science approaches in physiology. *Softwarex* **10**, 100287 (2019).
25. Bizzego, A. A data analytics framework for physiological signals from wearable devices. (University of Trento, 2017).
26. Eickhoff, S.B., *et al.* A new SPM toolbox for combining probabilistic cytoarchitectonic maps and functional imaging data. *Neuroimage* **25**, 1325-1335 (2005).
27. Gold, S., *et al.* Functional MRI statistical software packages: a comparative analysis. *Human brain mapping* **6**, 73-84 (1998).
